# Supplementary material for: Highly Active Heterogeneous Double Metal Cyanide Catalysts for Ring-Opening Polymerization of Cyclic Monomers
Source: Polymers (Basel). 2022 Jun 20;14(12):2507. doi: 10.3390/polym14122507 (PMC9227229; doi:10.3390/polym14122507)
Supplement: Supplementary file 1 [file polymers-14-02507-s001.zip › polymers-1755823-supplementary.pdf]

Supporting Information

Highly active heterogeneous double metal cyanide catalysts for ring-opening polymerization of cyclic monomers

*Chinh Hoang Tran, Min Woong Lee, Soo Jeong Lee, Jin Hyeok Choi, Eun Gyeong Lee, Ha Kyung Choi, and Il Kim**

School of Chemical Engineering, Pusan National University, Busandaehag-ro 63-2, Geumjeong-gu, Busan 46241, Republic of Korea.

* Correspondence: IK: ilkim@pusan.ac.kr

Table of Contents

1. Characterization of DMC catalysts S6

Figure S1 TGA curves of the DMC catalysts. S6

2. Polymerization of PO using DMC catalysts S7

Figure S2 ^{1}H NMR spectra (400 MHz, $CDCl_3$) of the crude reaction mixture of PO polymerization using PPG initiator and DMC-Ac catalyst. Conditions: catalyst amount = 100 mg, PO = 200 g, T_p = 115 °C. S7

Figure S3 ^{1}H NMR spectra (400 MHz, $CDCl_3$) of the crude reaction mixture of PO polymerization using PPG initiator and DMC-DMAc catalyst. Conditions: catalyst amount = 100 mg, PO = 200 g, T_p = 115 °C. S7

Figure S4 ^{1}H NMR spectra (400 MHz, $CDCl_3$) of the crude reaction mixture of PO polymerization using PPG initiator and DMC-DMF catalyst. Conditions: catalyst amount = 100 mg, PO = 200 g, T_p = 115 °C. S8

Figure S5 ^{1}H NMR spectra (400 MHz, $CDCl_3$) of the crude reaction mixture of PO polymerization using PPG initiator and DMC-DMSO catalyst. Conditions: catalyst amount = 100 mg, PO = 200 g, T_p = 115 °C. S8

Figure S6 ^{1}H NMR spectra (400 MHz, $CDCl_3$) of the crude reaction mixture of PO polymerization using PPG initiator and DMC-NMe catalyst. Conditions: catalyst amount = 100 mg, PO = 200 g, T_p = 115 °C. S9

Figure S7 ^{1}H NMR spectra (400 MHz, $CDCl_3$) of the crude reaction mixture of PO polymerization using PPG initiator and DMC-NMP catalyst. Conditions: catalyst amount = 100 mg, PO = 200 g, T_p = 115 °C. S9

3. Polymerization of CL using DMC catalysts S10

Figure S8 ^{1}H NMR spectra (400 MHz, $CDCl_3$) of the crude reaction mixture of CL polymerization using EG initiator and DMC-TBA catalyst. Conditions: catalyst amount = 10 mg ($[Zn]_0$ = 30 mM), $[CL]_0$ = 9 M, $[CL]_0/[EG]_0$ = 10, T_p = 160 °C. S10

Figure S9 ^{1}H NMR spectra (400 MHz, $CDCl_3$) of the crude reaction mixture of CL polymerization using EG initiator and DMC-DMAc catalyst. Conditions: catalyst amount = 10 mg ($[Zn]_0$ = 30 mM), $[CL]_0$ = 9 M, $[CL]_0/[EG]_0$ = 10, T_p = 160 °C. S10

Figure S10 ^{1}H NMR spectra (400 MHz, $CDCl_3$) of the crude reaction mixture of CL polymerization using EG initiator and DMC-DMF catalyst. Conditions: catalyst amount = 10 mg ($[Zn]_0$ = 30 mM), $[CL]_0$ = 9 M, $[CL]_0/[EG]_0$ = 10, T_p = 160 °C. S11

Figure S11 ^{1}H NMR spectra (400 MHz, $CDCl_3$) of the crude reaction mixture of CL polymerization using EG initiator and DMC-DMSO catalyst. Conditions: catalyst amount = 10 mg ($[Zn]_0$ = 30 mM), $[CL]_0$ = 9 M, $[CL]_0/[EG]_0$ = 10, T_p = 160 °C. S11

Figure S12 ^{1}H NMR spectra (400 MHz, $CDCl_3$) of the crude reaction mixture of CL polymerization using EG initiator and DMC-NMe catalyst. Conditions: catalyst amount = 10 mg ($[Zn]_0$ = 30 mM), $[CL]_0$ = 9 M, $[CL]_0/[EG]_0$ = 10, T_p = 160 °C. S12

Figure S13 ^{1}H NMR spectra (400 MHz, $CDCl_3$) of the crude reaction mixture of CL polymerization using EG initiator and DMC-NMP catalyst. Conditions: catalyst amount = 10 mg ($[Zn]_0$ = 30 mM), $[CL]_0$ = 9 M, $[CL]_0/[EG]_0$ = 10, T_p = 160 °C. S12

Figure S14 Leaching tests for the ROP of CL. The red dotted line indicates the conversion (X) of CL after separation of the solid catalyst. Reaction conditions: DMC-NMe = 10 mg ($[Zn]_0$ = 30 mM), $[CL]_0$ = 9 M, $[CL]_0/[EG]_0$ = 10, T_p = 160 °C. S13

Figure S15 MALDI-TOF MS spectrum of EG-PCL. Conditions: DMC-NMe = 10 mg ($[Zn]_0$ = 30 mM), $[CL]_0$ = 9 M, $[CL]_0/[EG]_0$ = 10, T_p = 160 °C........ S13

4. Polymerization of VL using DMC catalysts S14

Figure S16 ^{1}H NMR spectra (400 MHz, $CDCl_3$) of the crude reaction mixture of VL polymerization using EG initiator and DMC-1 catalyst. Conditions: catalyst amount = 10 mg ($[Zn]_0$ = 30 mM), $[VL]_0$ = 11 M, $[VL]_0/[EG]_0$ = 10, T_p = 160 °C. S14

Figure S17 ^{1}H NMR spectra (400 MHz, $CDCl_3$) of the crude reaction mixture of VL polymerization using EG initiator and DMC-TBA catalyst. Conditions: catalyst amount = 10 mg ($[Zn]_0$ = 30 mM), $[VL]_0$ = 11 M, $[VL]_0/[EG]_0$ = 10, T_p = 160 °C........ S14

Figure S18 ^{1}H NMR spectra (400 MHz, $CDCl_3$) of the crude reaction mixture of VL polymerization using EG initiator and DMC-DMAc catalyst. Conditions: catalyst amount = 10 mg ($[Zn]_0$ = 30 mM), $[VL]_0$ = 11 M, $[VL]_0/[EG]_0$ = 10, T_p = 160 °C. S15

Figure S19 ^{1}H NMR spectra (400 MHz, $CDCl_3$) of the crude reaction mixture of VL polymerization using EG initiator and DMC-DMF catalyst. Conditions: catalyst amount = 10 mg ($[Zn]_0$ = 30 mM), $[VL]_0$ = 11 M, $[VL]_0/[EG]_0$ = 10, T_p = 160 °C........ S15

Figure S20 ^{1}H NMR spectra (400 MHz, $CDCl_3$) of the crude reaction mixture of VL polymerization using EG initiator and DMC-DMSO catalyst. Conditions: catalyst amount = 10 mg ($[Zn]_0$ = 30 mM), $[VL]_0$ = 11 M, $[VL]_0/[EG]_0$ = 10, T_p = 160 °C. S16

Figure S21 ^{1}H NMR spectra (400 MHz, $CDCl_3$) of the crude reaction mixture of VL polymerization using EG initiator and DMC-NMe catalyst. Conditions: catalyst amount = 10 mg ($[Zn]_0$ = 30 mM), $[VL]_0$ = 11 M, $[VL]_0/[EG]_0$ = 10, T_p = 160 °C........ S16

Figure S22 ^{1}H NMR spectra (400 MHz, $CDCl_3$) of the crude reaction mixture of VL polymerization using EG initiator and DMC-NMP catalyst. Conditions: catalyst amount = 10 mg ($[Zn]_0$ = 30 mM), $[VL]_0$ = 11 M, $[VL]_0/[EG]_0$ = 10, T_p = 160 °C........ S17

Figure S23 GPC curves of the resultant PVLs produced by different $[VL]/[EG]$ ratios using DMC-NMe. Conditions: catalyst amount = 10 mg ($[Zn]_0$ = 30 mM), $[VL]_0$ = 11 M, T_p = 160 °C. S17

Figure S24 GPC curves of the resultant PVLs produced by different $[VL]/[GL]$ ratios using DMC-NMe. Conditions: catalyst amount = 10 mg ($[Zn]_0$ = 30 mM), $[VL]_0$ = 11 M, T_p = 160 °C. S18

Figure S25 MALDI-TOF MS spectrum of EG-PVL. Conditions: DMC-NMe = 10 mg ($[Zn]_0$ = 30 mM), $[VL]_0$ = 11 M, $[VL]_0/[EG]_0$ = 10, T_p = 160 °C. S18

Figure S26 MALDI-TOF MS spectrum of GL-PVL. Conditions: DMC-NMe = 10 mg ($[Zn]_0$ = 30 mM), $[VL]_0$ = 11 M, $[VL]_0/[GL]_0$ = 10, T_p = 160 °C. S19

5. Copolymerization of PO and lactones S20

Figure S27 ^{1}H NMR spectra (400 MHz, $CDCl_3$) of the PPO-PCL copolymer obtained by copolymerization of PO and CL using DMC-NMe catalyst. Conditions: catalyst amount = 2 mg ($[Zn]_0$ = 6 mM), PO = 1.8 mmol, CL = 18 mmol, T_p = 140 °C, t = 8 h. S20

Figure S28 ^{1}H NMR spectra (400 MHz, $CDCl_3$) of the PPO-PVL copolymer obtained by copolymerization of PO and VL using DMC-NMe catalyst. Conditions: catalyst amount = 2 mg ($[Zn]_0$ = 6 mM), PO = 1.8 mmol, VL = 18 mmol, T_p = 140 °C, t = 8 h. S20

Figure S29 GPC curves of the resultant copolymers obtained by copolymerization of PO and lactone using DMC-NMe. catalyst amount = 2 mg ($[Zn]_0$ = 6 mM), PO = 1.8 mmol, lactone = 18 mmol, T_p = 140 °C, t = 8 h. S21

6. Kinetic studies of the ROP of VL S22

Figure S30. In situ ^{1}H NMR (400 MHz, CD_3OD) spectra of the ROP of VL at 130 °C. Conditions: DMC-NMe = 10 mg, $[VL]_0$ = 11 M, $[VL]_0/[GL]_0$ = 10. Dashed lines denote the monomer signals. S23

Figure S31 In situ ^{1}H NMR (400 MHz, CD_3OD) spectra of the ROP of VL at 140 °C. Conditions: DMC-NMe = 10 mg, $[VL]_0$ = 11 M, $[CL]_0/[GL]_0$ = 10. Dashed lines denote the monomer signals. S24

Figure S32 In situ ^{1}H NMR (400 MHz, CD_3OD) spectra of the ROP of VL at 150 °C. Conditions: DMC-NMe = 10 mg, $[VL]_0$ = 11 M, $[CL]_0/[GL]_0$ = 10. Dashed lines denote the monomer signals. S24

Figure S33 In situ ^{1}H NMR (400 MHz, CD_3OD) spectra of the ROP of VL at 160 °C. Conditions: DMC-NMe = 10 mg, $[VL]_0$ = 11 M, $[CL]_0/[GL]_0$ = 10. Dashed lines denote the monomer signals. S25

Figure S34 In situ ^{1}H NMR (400 MHz, CD_3OD) spectra of the ROP of VL at 160 °C. Conditions: DMC-NMe = 10 mg, $[VL]_0$ = 11 M, $[CL]_0/[GL]_0$ = 5. Dashed lines denote the monomer signals. S25

Figure S35 In situ ^{1}H NMR (400 MHz, CD_3OD) spectra of the ROP of VL at 160 °C. Conditions: DMC-NMe = 10 mg, $[VL]_0$ = 11 M, $[CL]_0/[GL]_0$ = 20. Dashed lines denote the monomer signals. S26

Figure S36 In situ ^{1}H NMR (400 MHz, CD_3OD) spectra of the ROP of VL at 160 °C. Conditions: DMC-NMe = 10 mg, $[VL]_0$ = 11 M, $[CL]_0/[GL]_0$ = 50. Dashed lines denote the monomer signals. S26

Figure S37 (a)–(d) Plots of monomer conversion versus time obtained at different temperatures, (e) First-order time–conversion plots, and (f) Arrhenius plot of the ROP of VL. Conditions: DMC-NMe = 10 mg, $[VL]_0$ = 11 M, $[VL]_0/[GL]_0$ = 10. .... S27

Figure S38 (a) First-order time–conversion plots of the ROP of VL at 160 °C using different amounts of initiator and (b) dependence of the k_{app} on the concentration of initiator [*GL*]. Conditions: DMC-NMe = 10 mg, $[VL]_0$ = 11 M, T_p = 160 °C. S28

Figure S39 (a) GPC curves of PVL collected at different intervals, plots of M_n and $Đ$ versus (b) monomer conversion and (c) polymerization time. Conditions: DMC-NMe = 10 mg, $[VL]_0$ = 11 M, $[VL]_0/[GL]_0$ = 20, T_p = 160 °C. S28

7. Supplementary tables S30

Table S1 Summary of the XPS results of Zn 2p spin-orbital components S30

Table S2 Results for the ROP of VL by DMC-NMe using various $[VL]_0/[I]_0$ ratios S30

Table S3 Results for the copolymerization of PO and lactone using DMC-NMe............. S30

1. Characterization of DMC catalysts

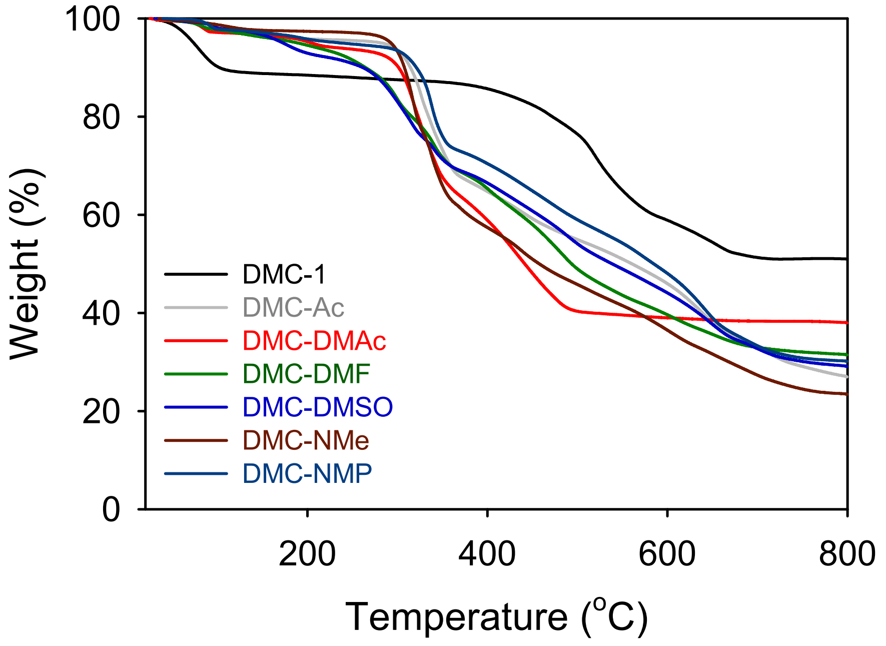

Figure S1 TGA curves of the DMC catalysts.

2. Polymerization of PO using DMC catalysts

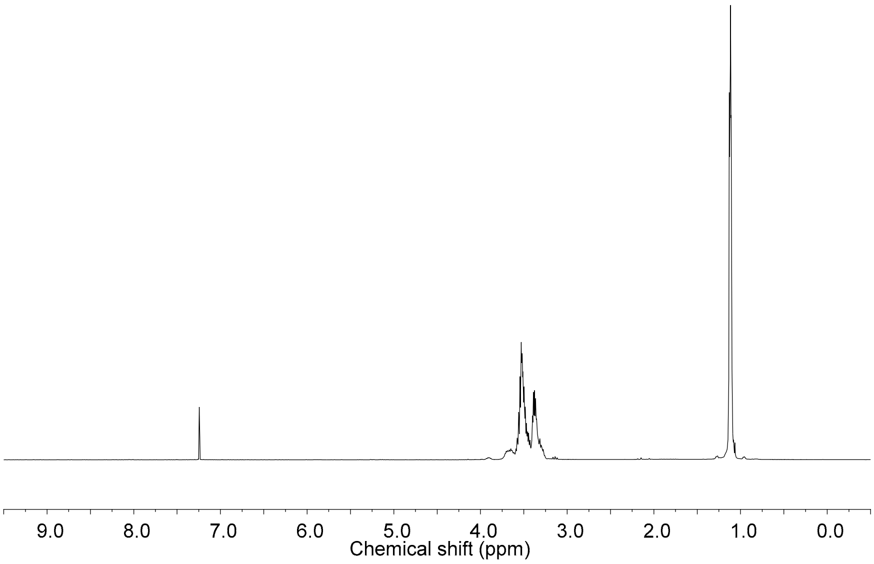

Figure S2 ^1H NMR spectra (400 MHz, $CDCl_3$) of the crude reaction mixture of PO polymerization using PPG initiator and DMC-Ac catalyst. Conditions: catalyst amount = 100 mg, PO = 200 g, T_p = 115 °C.

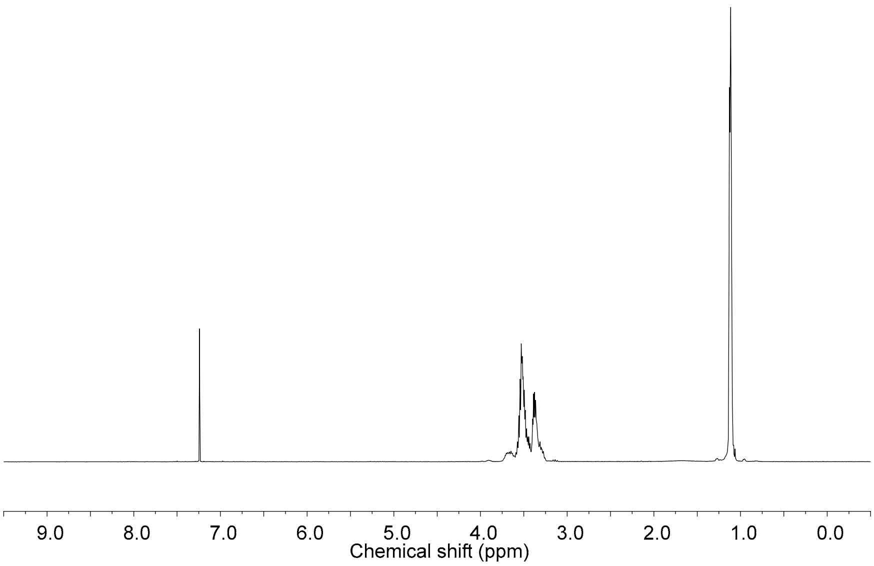

Figure S3 ^1H NMR spectra (400 MHz, $CDCl_3$) of the crude reaction mixture of PO polymerization using PPG initiator and DMC-DMAc catalyst. Conditions: catalyst amount = 100 mg, PO = 200 g, T_p = 115 °C.

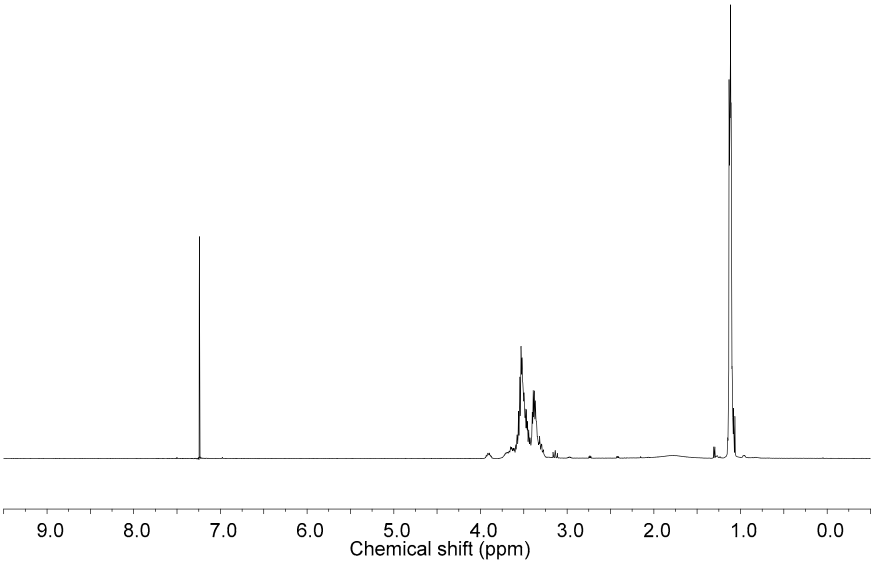

Figure S4 ^{1}H NMR spectra (400 MHz, $CDCl_3$) of the crude reaction mixture of PO polymerization using PPG initiator and DMC-DMF catalyst. Conditions: catalyst amount = 100 mg, PO = 200 g, T_p = 115 °C.

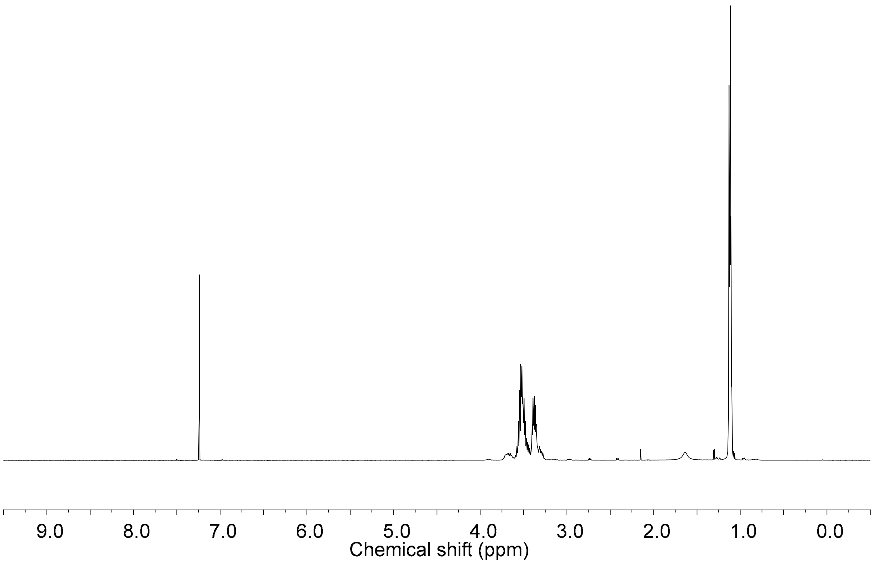

Figure S5 ^{1}H NMR spectra (400 MHz, $CDCl_3$) of the crude reaction mixture of PO polymerization using PPG initiator and DMC-DMSO catalyst. Conditions: catalyst amount = 100 mg, PO = 200 g, T_p = 115 °C.

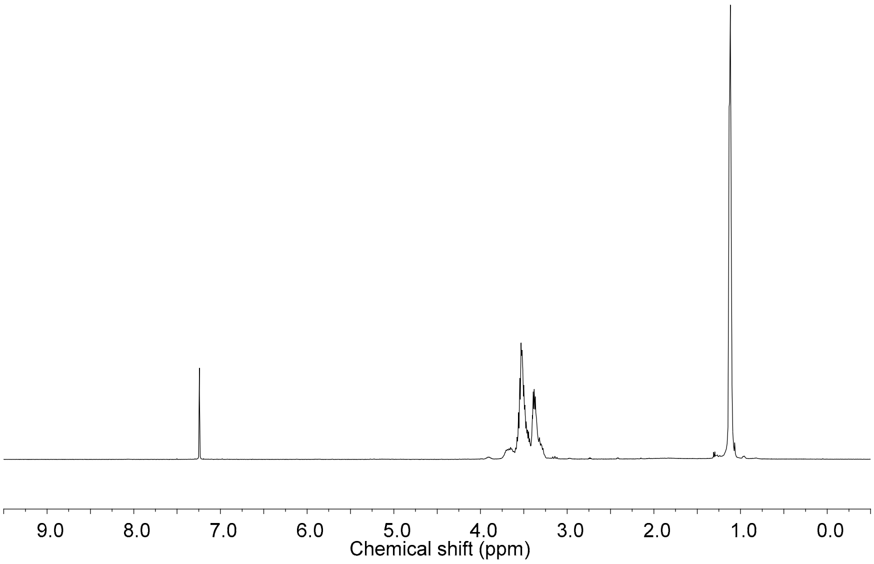

Figure S6 ^{1}H NMR spectra (400 MHz, $CDCl_3$) of the crude reaction mixture of PO polymerization using PPG initiator and DMC-NMe catalyst. Conditions: catalyst amount = 100 mg, PO = 200 g, T_p = 115 °C.

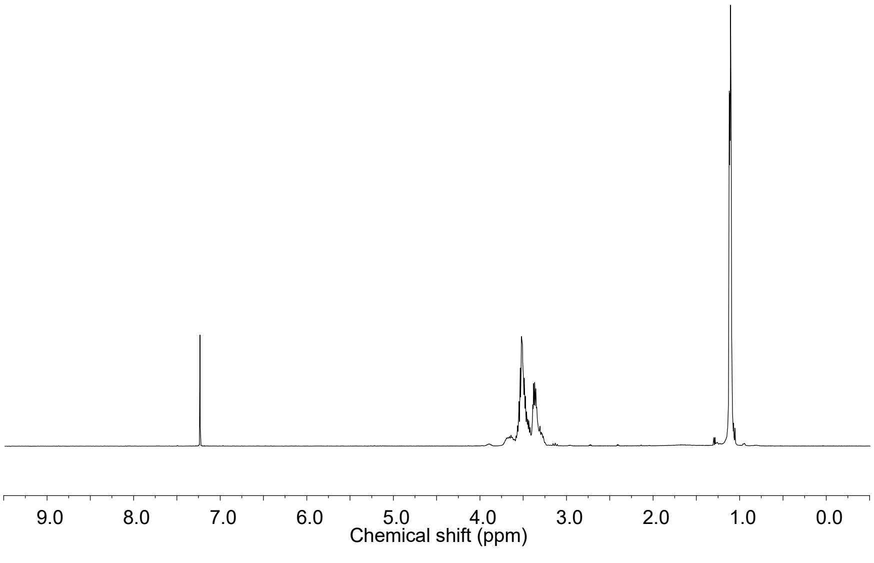

Figure S7 ^{1}H NMR spectra (400 MHz, $CDCl_3$) of the crude reaction mixture of PO polymerization using PPG initiator and DMC-NMP catalyst. Conditions: catalyst amount = 100 mg, PO = 200 g, T_p = 115 °C.

3. Polymerization of CL using DMC catalysts

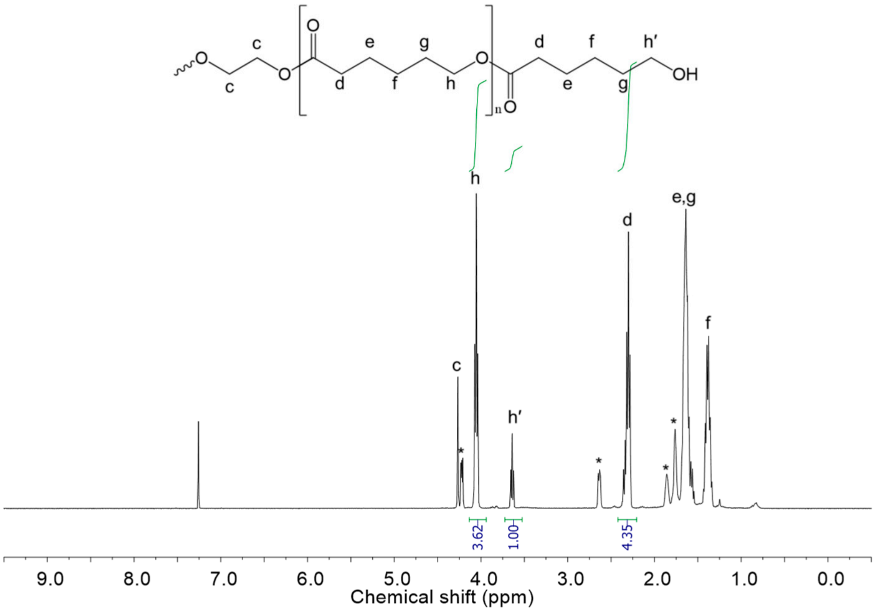

Figure S8 ^{1}H NMR spectra (400 MHz, $CDCl_3$) of the crude reaction mixture of CL polymerization using EG initiator and DMC-TBA catalyst. Conditions: catalyst amount = 10 mg ($[Zn]_0$ = 30 mM), $[CL]_0$ = 9 M, $[CL]_0/[EG]_0$ = 10, T_p = 160 °C.

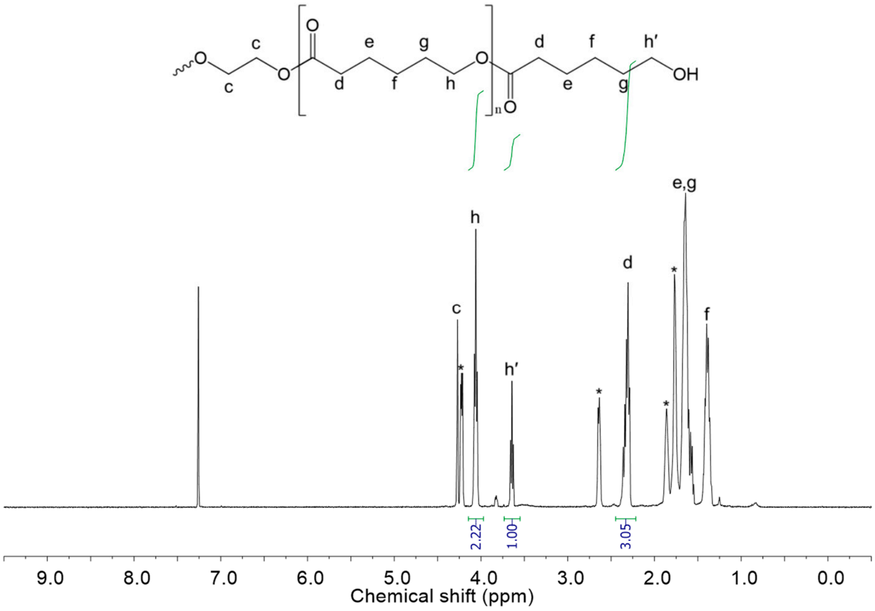

Figure S9 ^{1}H NMR spectra (400 MHz, $CDCl_3$) of the crude reaction mixture of CL polymerization using EG initiator and DMC-DMAc catalyst. Conditions: catalyst amount = 10 mg ($[Zn]_0$ = 30 mM), $[CL]_0$ = 9 M, $[CL]_0/[EG]_0$ = 10, T_p = 160 °C.

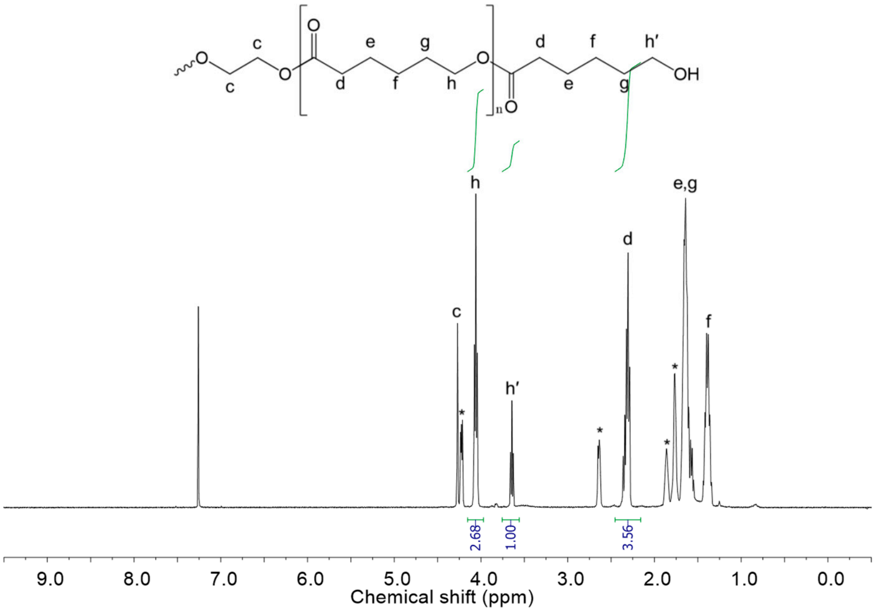

Figure S10 ^{1}H NMR spectra (400 MHz, $CDCl_3$) of the crude reaction mixture of CL polymerization using EG initiator and DMC-DMF catalyst. Conditions: catalyst amount = 10 mg ($[Zn]_0$ = 30 mM), $[CL]_0$ = 9 M, $[CL]_0/[EG]_0$ = 10, T_p = 160 °C.

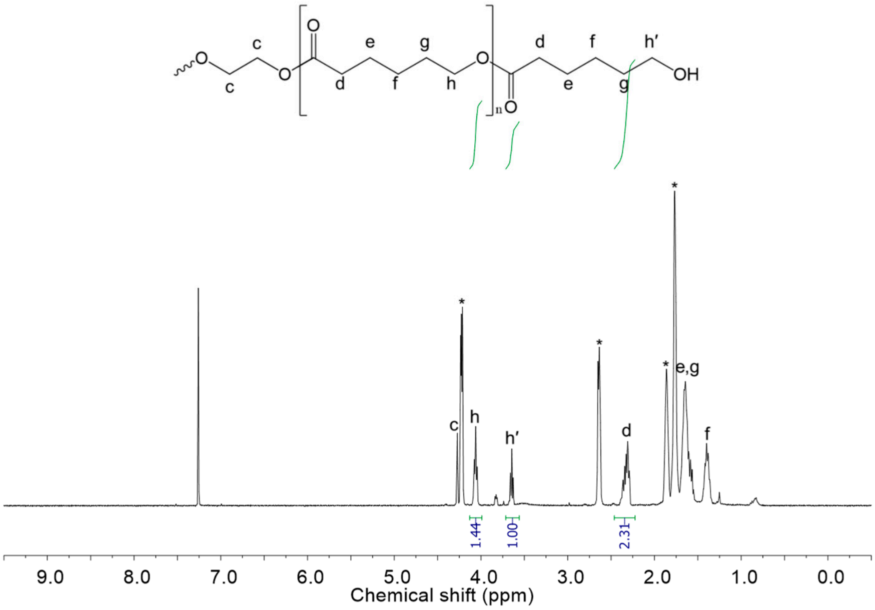

Figure S11 ^{1}H NMR spectra (400 MHz, $CDCl_3$) of the crude reaction mixture of CL polymerization using EG initiator and DMC-DMSO catalyst. Conditions: catalyst amount = 10 mg ($[Zn]_0$ = 30 mM), $[CL]_0$ = 9 M, $[CL]_0/[EG]_0$ = 10, T_p = 160 °C.

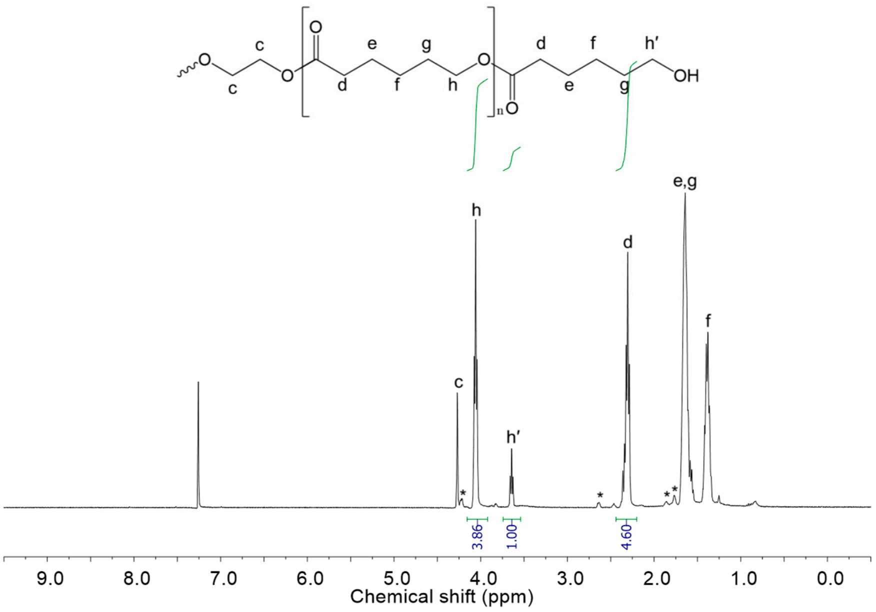

Figure S12 ^{1}H NMR spectra (400 MHz, $CDCl_3$) of the crude reaction mixture of CL polymerization using EG initiator and DMC-NMe catalyst. Conditions: catalyst amount = 10 mg ($[Zn]_0$ = 30 mM), $[CL]_0$ = 9 M, $[CL]_0/[EG]_0$ = 10, T_p = 160 °C.

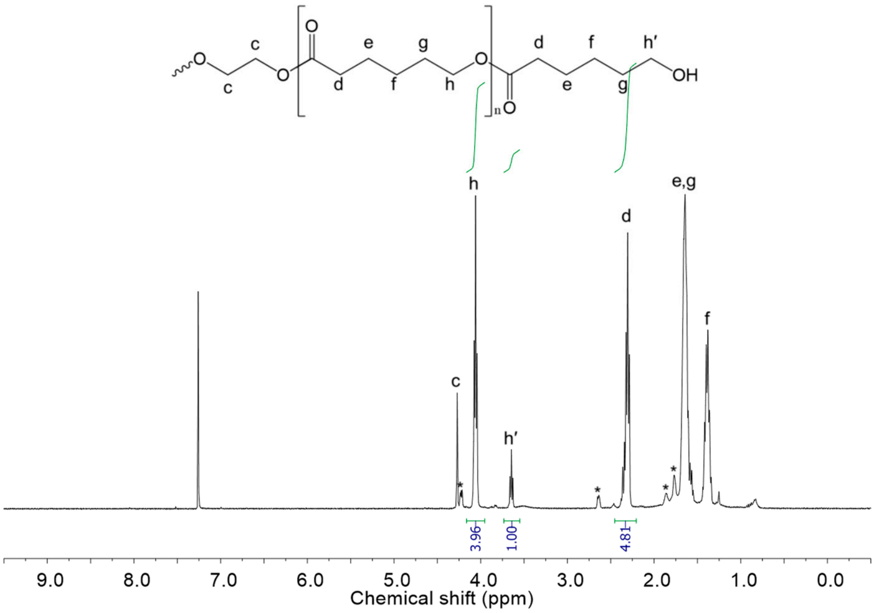

Figure S13 ^{1}H NMR spectra (400 MHz, $CDCl_3$) of the crude reaction mixture of CL polymerization using EG initiator and DMC-NMP catalyst. Conditions: catalyst amount = 10 mg ($[Zn]_0$ = 30 mM), $[CL]_0$ = 9 M, $[CL]_0/[EG]_0$ = 10, T_p = 160 °C.

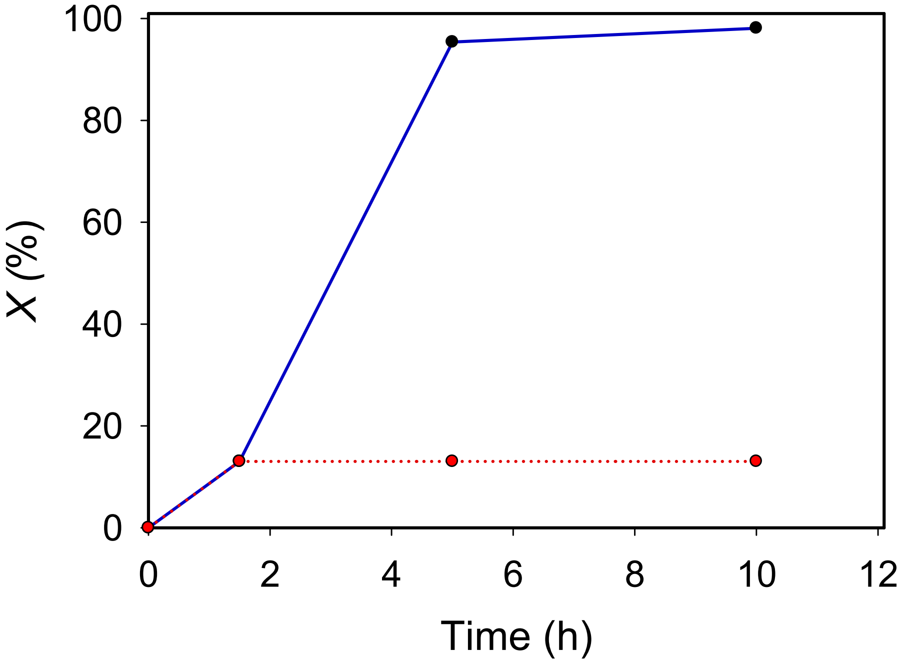

Figure S14 Leaching tests for the ROP of CL. The red dotted line indicates the conversion (*X*) of CL after separation of the solid catalyst. Reaction conditions: DMC-NMe = 10 mg ($[Zn]_0$ = 30 mM), $[CL]_0$ = 9 M, $[CL]_0/[EG]_0$ = 10, T_p = 160 °C.

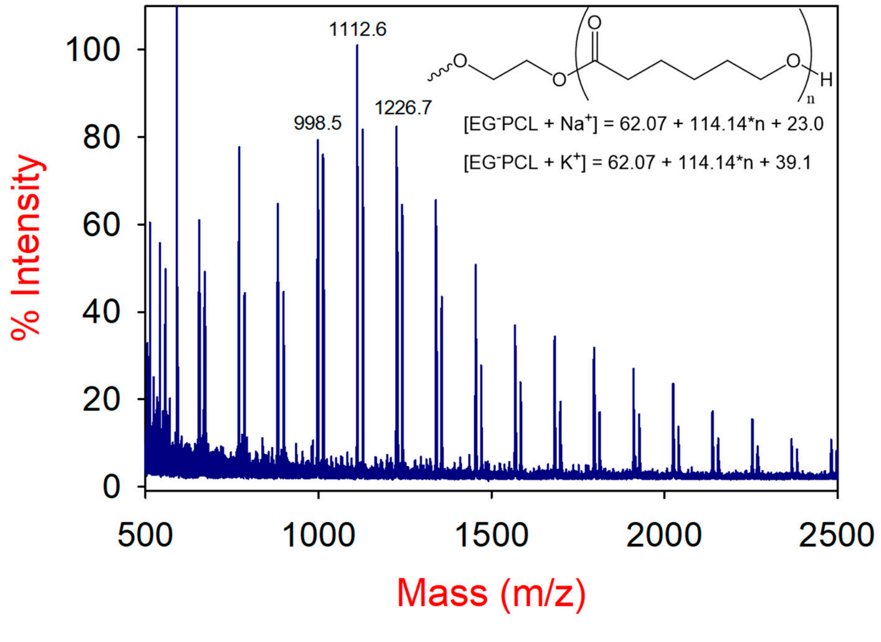

Figure S15 MALDI-TOF MS spectrum of EG-PCL. Conditions: DMC-NMe = 10 mg ($[Zn]_0$ = 30 mM), $[CL]_0$ = 9 M, $[CL]_0/[EG]_0$ = 10, T_p = 160 °C.

4. Polymerization of VL using DMC catalysts

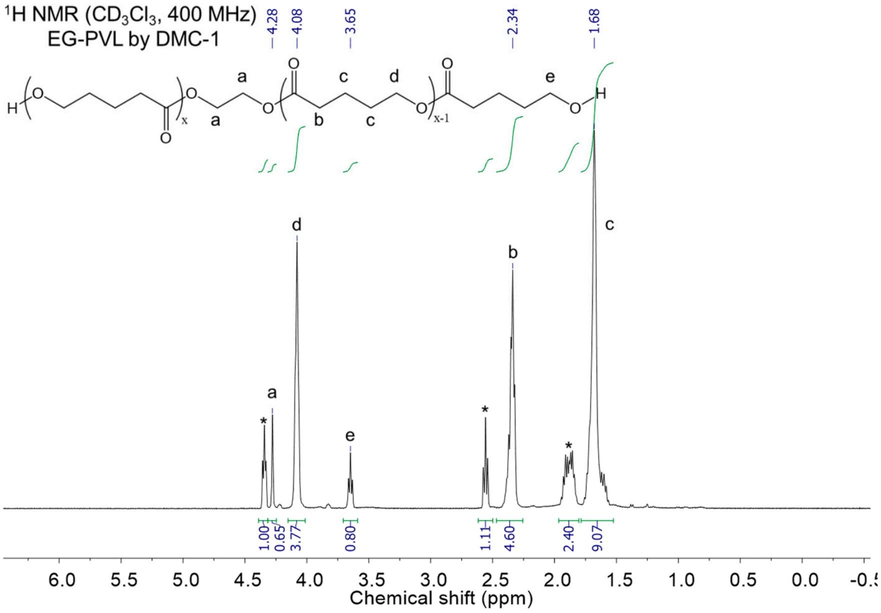

Figure S16 ^{1}H NMR spectra (400 MHz, $CDCl_3$) of the crude reaction mixture of VL polymerization using EG initiator and DMC-1 catalyst. Conditions: catalyst amount = 10 mg ($[Zn]_0$ = 30 mM), $[VL]_0$ = 11 M, $[VL]_0/[EG]_0$ = 10, T_p = 160 °C.

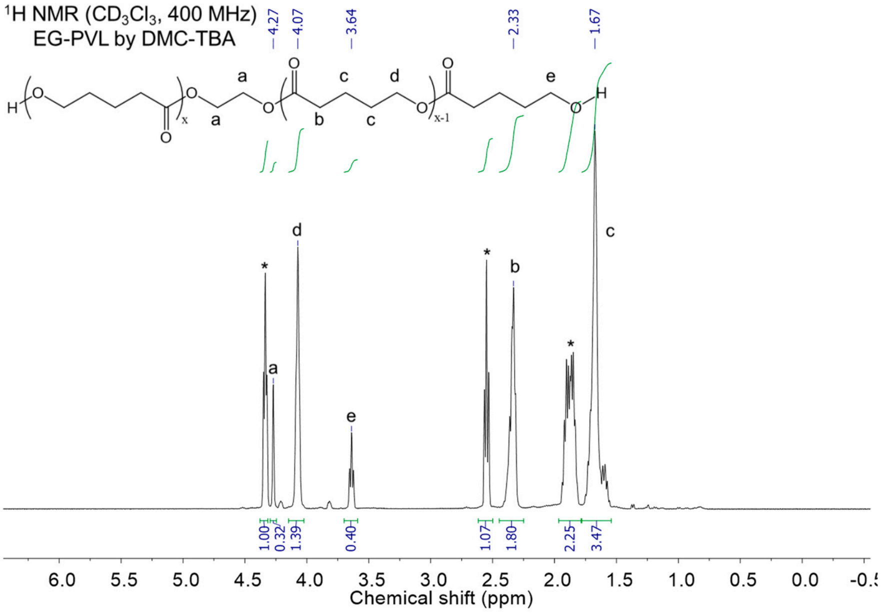

Figure S17 ^{1}H NMR spectra (400 MHz, $CDCl_3$) of the crude reaction mixture of VL polymerization using EG initiator and DMC-TBA catalyst. Conditions: catalyst amount = 10 mg ($[Zn]_0$ = 30 mM), $[VL]_0$ = 11 M, $[VL]_0/[EG]_0$ = 10, T_p = 160 °C.

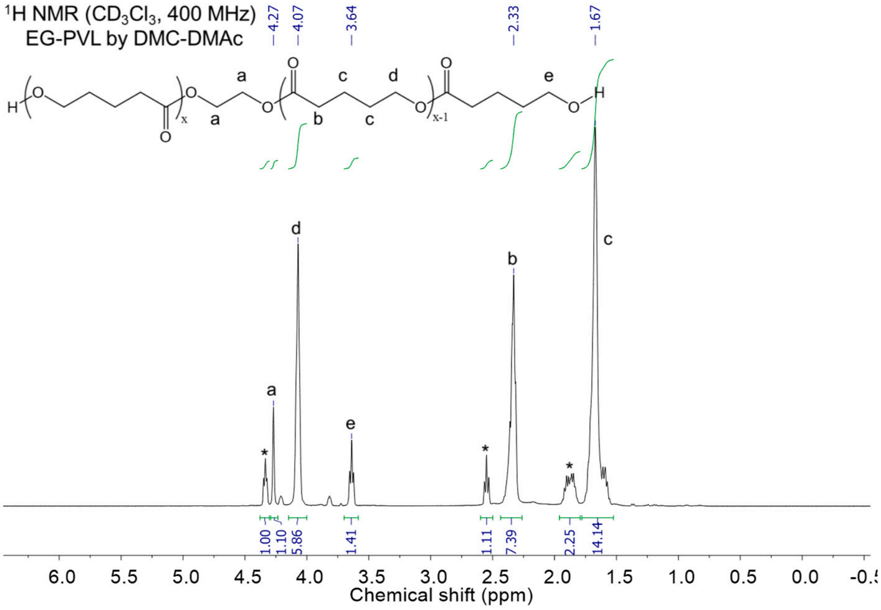

Figure S18 ^{1}H NMR spectra (400 MHz, CDCl$_3$) of the crude reaction mixture of VL polymerization using EG initiator and DMC-DMAc catalyst. Conditions: catalyst amount = 10 mg ($[Zn]_0$ = 30 mM), $[VL]_0$ = 11 M, $[VL]_0/[EG]_0$ = 10, T_p = 160 °C.

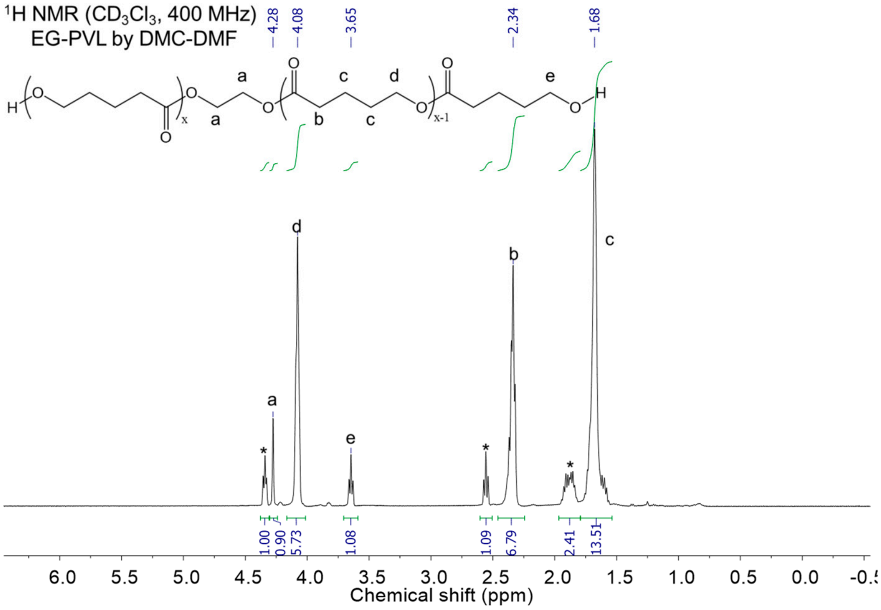

Figure S19 ^{1}H NMR spectra (400 MHz, CDCl$_3$) of the crude reaction mixture of VL polymerization using EG initiator and DMC-DMF catalyst. Conditions: catalyst amount = 10 mg ($[Zn]_0$ = 30 mM), $[VL]_0$ = 11 M, $[VL]_0/[EG]_0$ = 10, T_p = 160 °C.

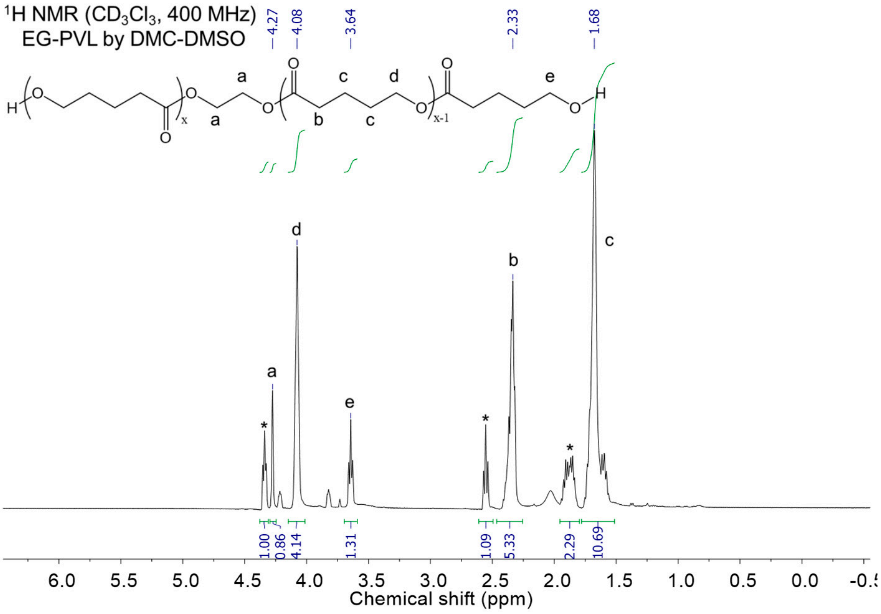

Figure S20 ^{1}H NMR spectra (400 MHz, $CDCl_3$) of the crude reaction mixture of VL polymerization using EG initiator and DMC-DMSO catalyst. Conditions: catalyst amount = 10 mg ($[Zn]_0$ = 30 mM), $[VL]_0$ = 11 M, $[VL]_0/[EG]_0$ = 10, T_p = 160 °C.

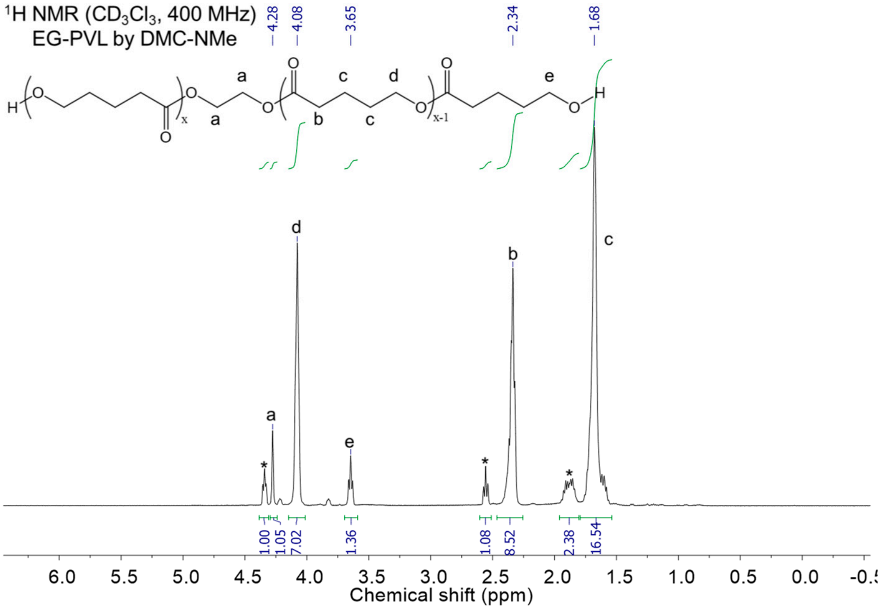

Figure S21 ^{1}H NMR spectra (400 MHz, $CDCl_3$) of the crude reaction mixture of VL polymerization using EG initiator and DMC-NMe catalyst. Conditions: catalyst amount = 10 mg ($[Zn]_0$ = 30 mM), $[VL]_0$ = 11 M, $[VL]_0/[EG]_0$ = 10, T_p = 160 °C.

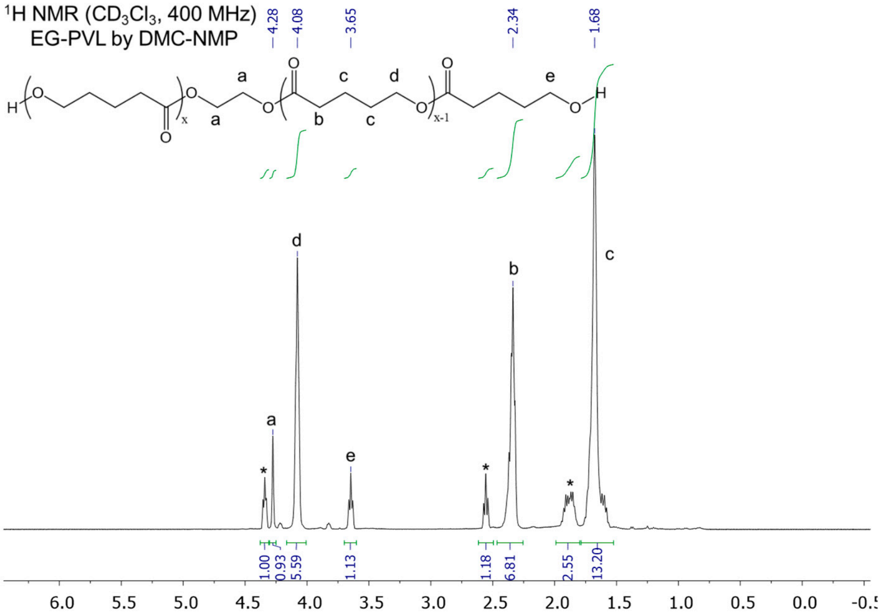

Figure S22 ^{1}H NMR spectra (400 MHz, $CDCl_3$) of the crude reaction mixture of VL polymerization using EG initiator and DMC-NMP catalyst. Conditions: catalyst amount = 10 mg ($[Zn]_0$ = 30 mM), $[VL]_0$ = 11 M, $[VL]_0/[EG]_0$ = 10, T_p = 160 °C.

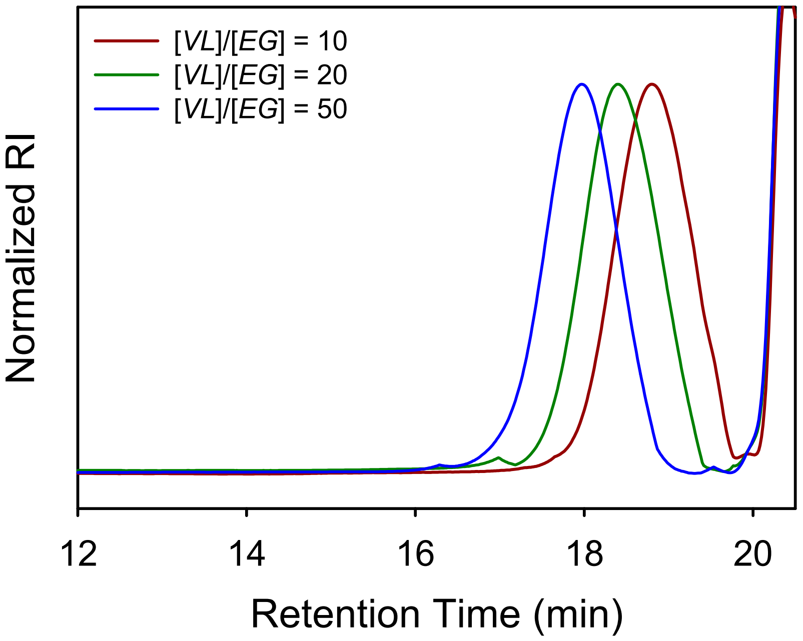

Figure S23 GPC curves of the resultant PVLs produced by different $[VL]/[EG]$ ratios using DMC-NMe. Conditions: catalyst amount = 10 mg ($[Zn]_0$ = 30 mM), $[VL]_0$ = 11 M, T_p = 160 °C.

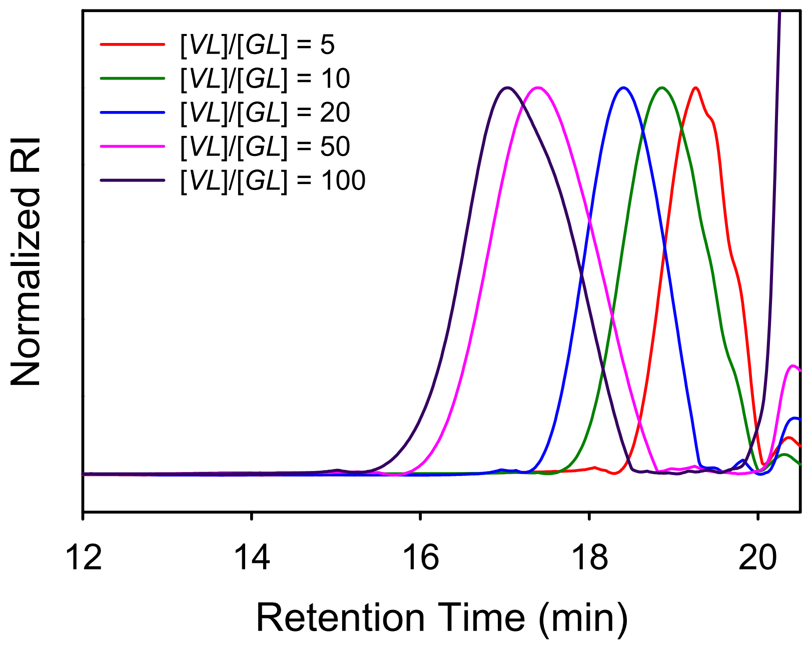

Figure S24 GPC curves of the resultant PVLs produced by different [*VL*]/[*GL*] ratios using DMC-NMe. Conditions: catalyst amount = 10 mg ([*Zn*]$_0$ = 30 mM), [*VL*]$_0$ = 11 M, T_p = 160 °C.

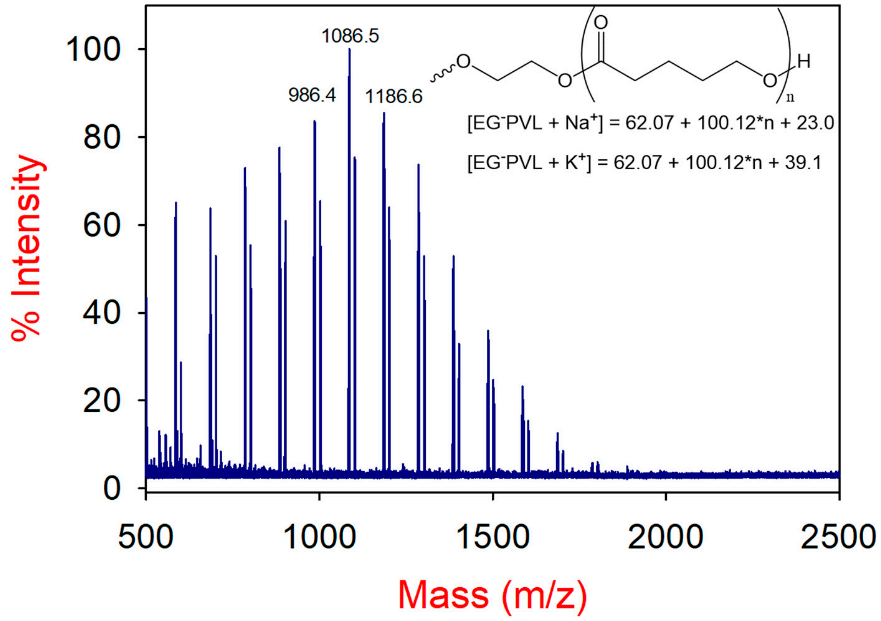

Figure S25 MALDI-TOF MS spectrum of EG-PVL. Conditions: DMC-NMe = 10 mg ([*Zn*]$_0$ = 30 mM), [*VL*]$_0$ = 11 M, [*VL*]$_0$/[*EG*]$_0$ = 10, T_p = 160 °C.

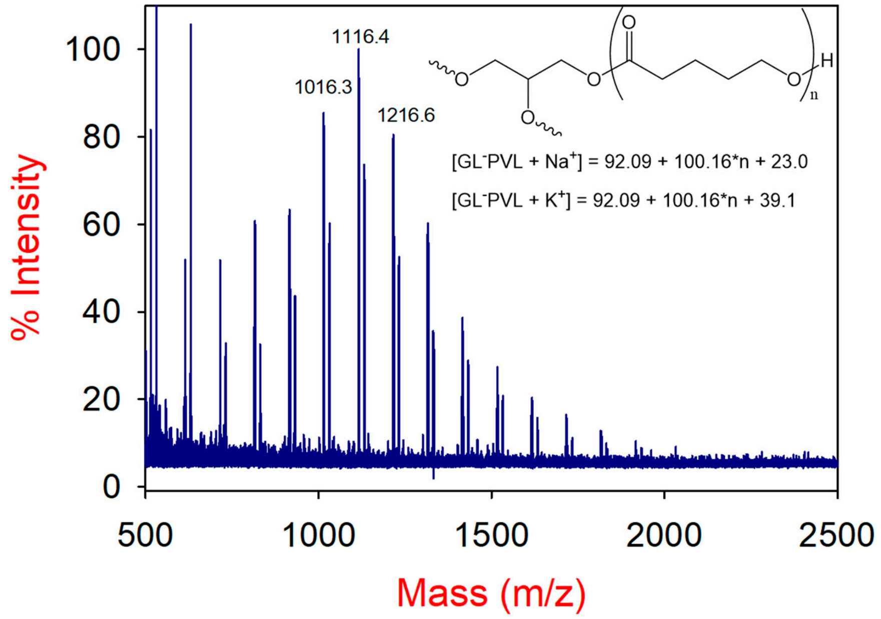

Figure S26 MALDI-TOF MS spectrum of GL-PVL. Conditions: DMC-NMe = 10 mg ($[Zn]_0$ = 30 mM), $[VL]_0$ = 11 M, $[VL]_0/[GL]_0$ = 10, T_p = 160 °C.

5. Copolymerization of PO and lactones

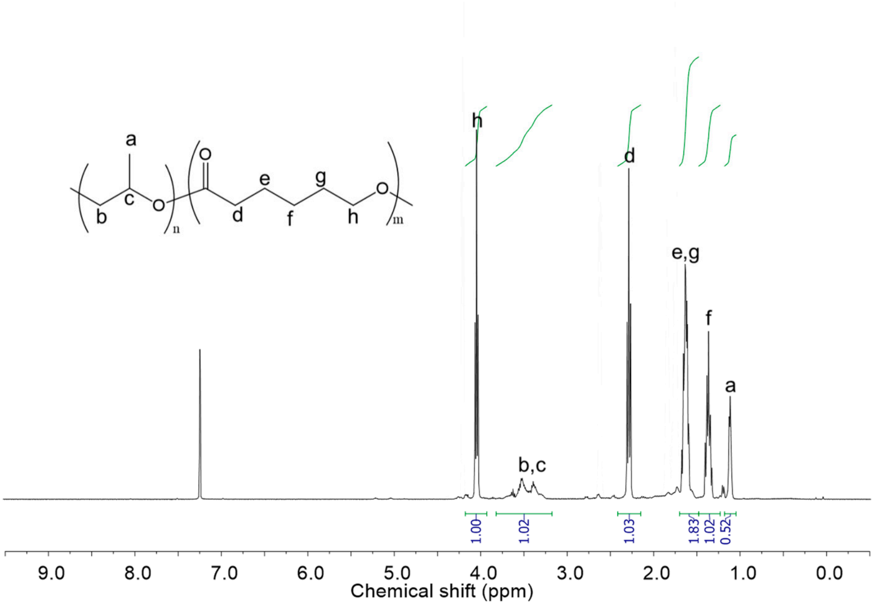

Figure S27 ^{1}H NMR spectra (400 MHz, $CDCl_3$) of the PPO-PCL copolymer obtained by copolymerization of PO and CL using DMC-NMe catalyst. Conditions: catalyst amount = 2 mg ($[Zn]_0$ = 6 mM), PO = 1.8 mmol, CL = 18 mmol, T_p = 140 °C, t = 8 h.

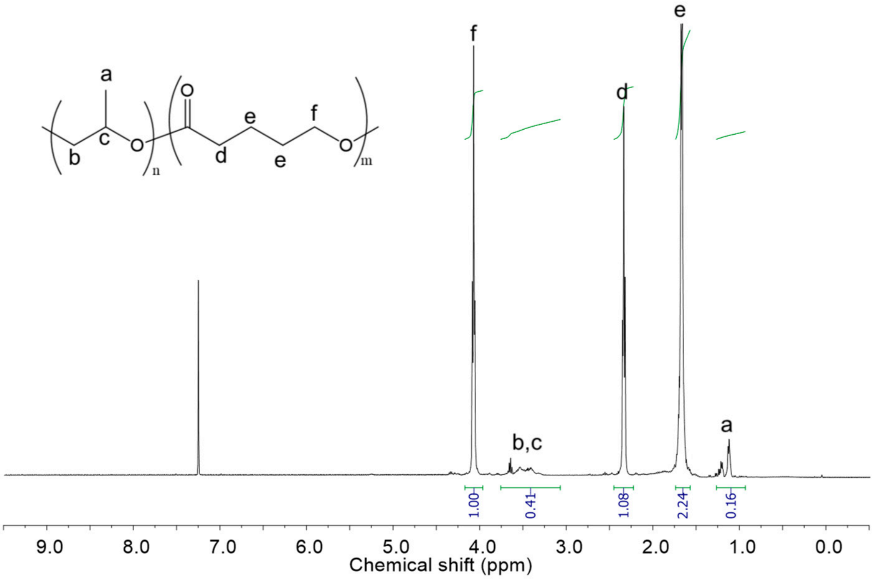

Figure S28 ^{1}H NMR spectra (400 MHz, $CDCl_3$) of the PPO-PVL copolymer obtained by copolymerization of PO and VL using DMC-NMe catalyst. Conditions: catalyst amount = 2 mg ($[Zn]_0$ = 6 mM), PO = 1.8 mmol, VL = 18 mmol, T_p = 140 °C, t = 8 h.

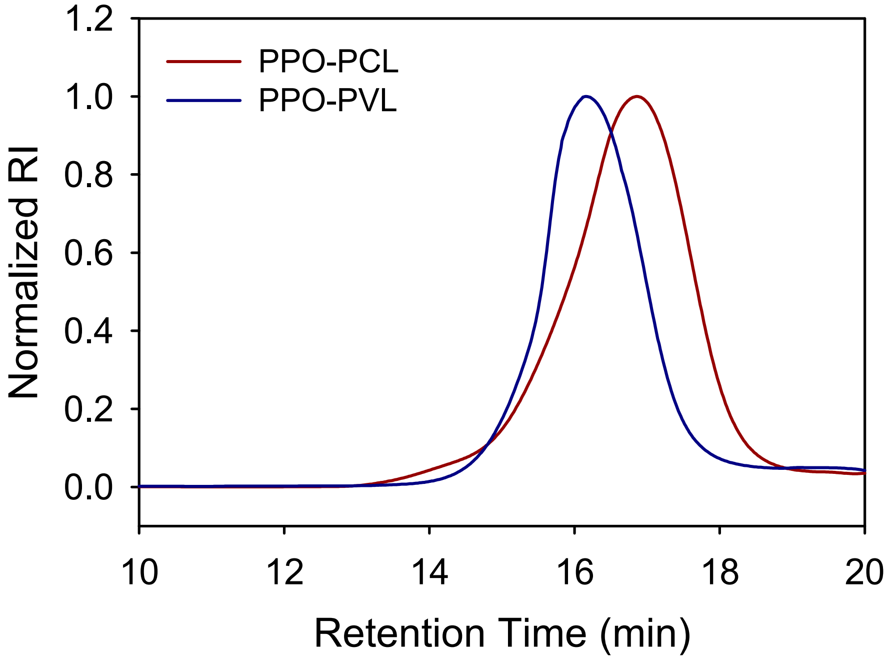

Figure S29 GPC curves of the resultant copolymers obtained by copolymerization of PO and lactone using DMC-NMe. catalyst amount = 2 mg ([*Zn*]$_0$ = 6 mM), PO = 1.8 mmol, lactone = 18 mmol, T_p = 140 °C, t = 8 h.

6. Kinetic studies of the ROP of VL

To study the reaction kinetics, the ROP of VL was further investigated at various polymerization conditions using a GL initiator and DMC-NMe catalysts. The polymerizations were firstly conducted at different temperatures from 130 to 160 °C, in which the monomer conversion and MW evolution were monitored during the reaction. The ^{1}H NMR spectra collected at different intervals clearly indicate the gradual decrease of the monomer signals at 1.87, 2.56, and 4.35 ppm along with the growth of polyester backbone signals at 1.68, 2.34, 3.65 and 4.08 ppm with the reaction time (Figs. S11–S14).

The monomer conversion versus reaction time at 130–160 °C was plotted to determine the reaction rate (Figure S18(a)–(d)). The linear plots of $\ln([VL]_0/[VL])$ versus time indicate that the polymerization of VL is pseudo-first-order dependence on the monomer concentration and the polymerizations proceed without induction periods (Figure S18(e)). The plot of $\ln k_{app}$ versus $1/T$ was used to determine the overall activation energy (E_a) using equation (1).

$$k_{\mathrm{app}} = \mathrm{A}e^{(-\mathrm{E_a}/\mathrm{RT})} \qquad \ln k_{\mathrm{app}} - \frac{E_{\mathrm{a}}}{\mathrm{RT}} + \ln \mathrm{A} \qquad (1)$$

Here, k_{app} is apparent rate constant, T is the absolute reaction temperature (°K).

The dependence of the apparent rate constant k_{app} on the concentration of initiator was further investigated at 160 °C using different $[VL]_0/[GL]_0$ ratios (Figs. S15–S17). The linear plots of $\ln(k_{\mathrm{app}})$ versus $\ln[GL]$ indicate that the polymerization was also first-order with respect to the initiator concentration (Figure S19). Hence, the rate equation can be described as equation (2).

$$-\frac{\mathrm{d}[VL]}{\mathrm{dt}} = k_{\mathrm{p}}[VL][GL] \qquad (2)$$

Here, k_{p} is the propagation rate constant and $k_{\mathrm{app}} = k_{\mathrm{p}}[GL]$. Equation (2) can also be presented as equation (3).

$$-\frac{d[VL]}{dt} = k_{app}[VL] \quad (3)$$

This rate law is consistent to the coordination polymerization mechanism at the Zn site. In addition, GPC curves of the GL-PVL sampled during polymerization indicate that the MW increases monotonically with respect to the monomer conversion, which is frequently observed in a chain-growth polymerization (Figure S20). The *Đ* values remain narrow during the polymerization (1.01–1.33); however, significantly broadened when the monomer conversion exceeded 90% (1.63-1.91), most probably due to intermolecular transesterification reactions.

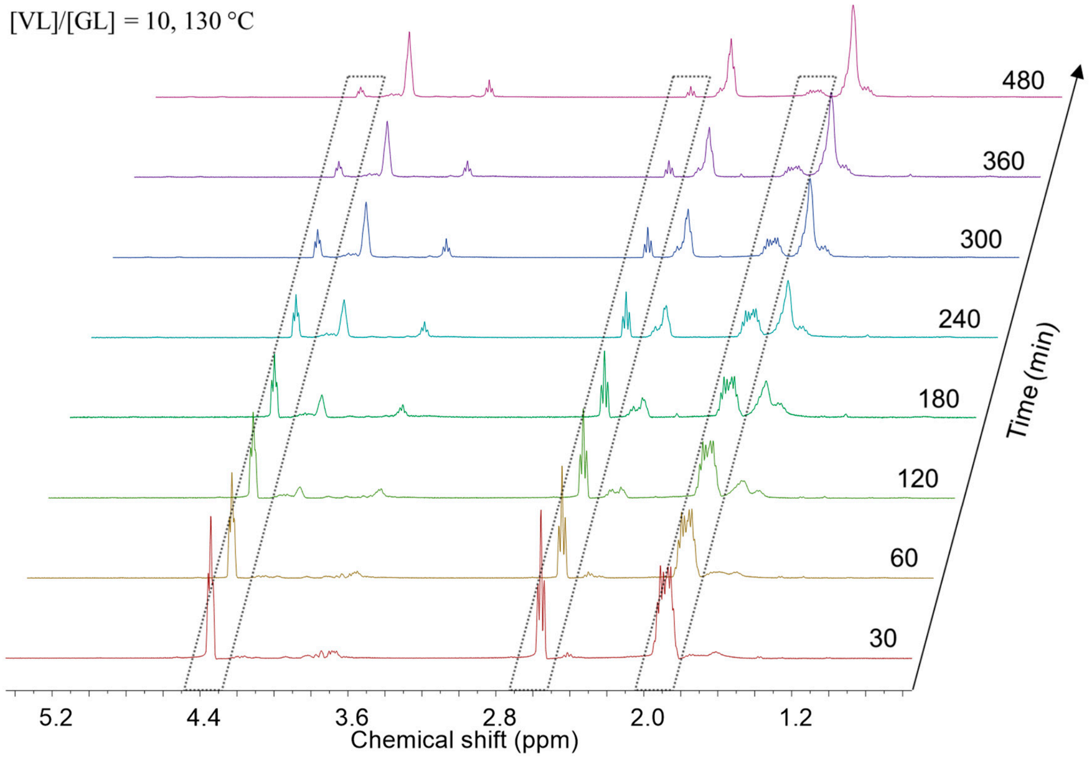

Figure S30. In situ ^1H NMR (400 MHz, CD_3OD) spectra of the ROP of VL at 130 °C. Conditions: DMC-NMe = 10 mg, $[VL]_0$ = 11 M, $[VL]_0/[GL]_0$ = 10. Dashed lines denote the monomer signals.

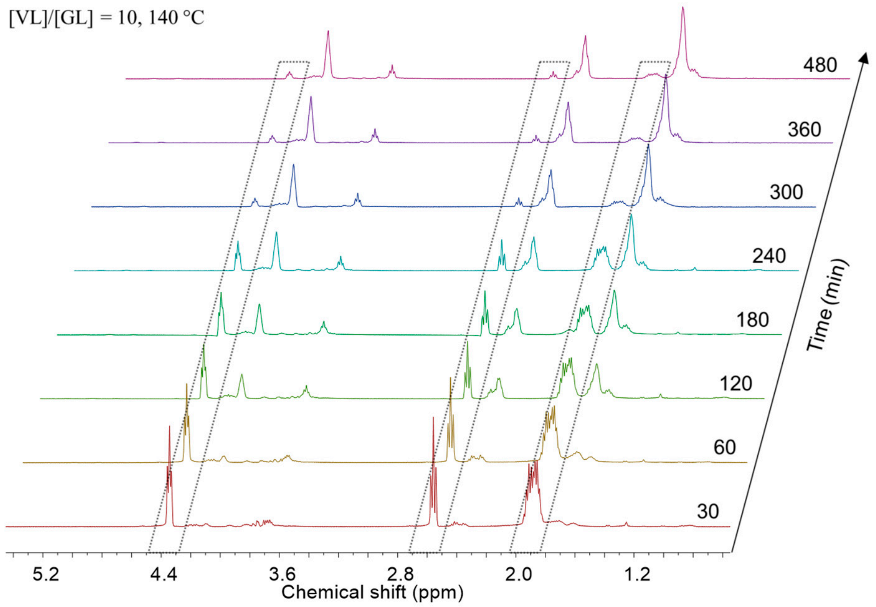

Figure S31 In situ ^{1}H NMR (400 MHz, CD_3OD) spectra of the ROP of VL at 140 °C. Conditions: DMC-NMe = 10 mg, $[VL]_0$ = 11 M, $[VL]_0/[GL]_0$ = 10. Dashed lines denote the monomer signals.

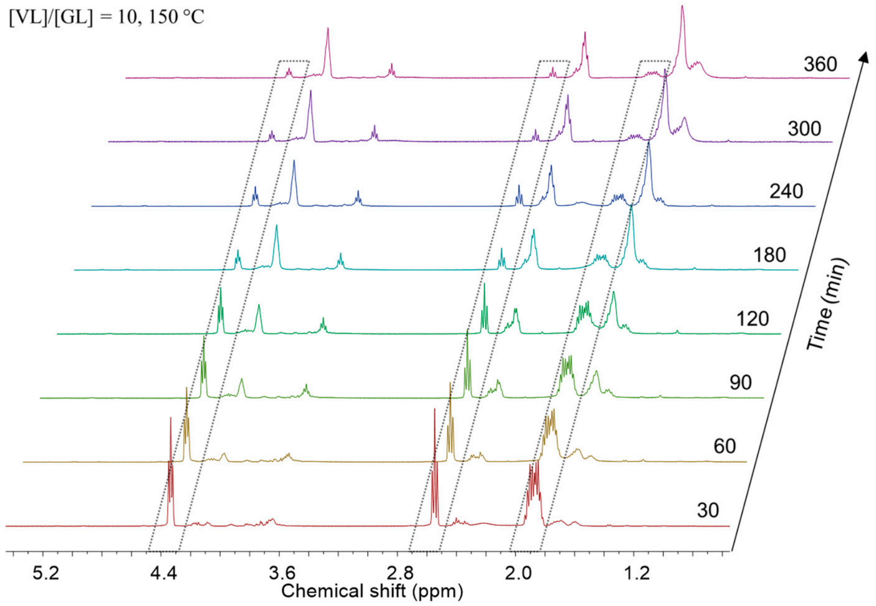

Figure S32 In situ ^{1}H NMR (400 MHz, CD_3OD) spectra of the ROP of VL at 150 °C. Conditions: DMC-NMe = 10 mg, $[VL]_0$ = 11 M, $[VL]_0/[GL]_0$ = 10. Dashed lines denote the monomer signals.

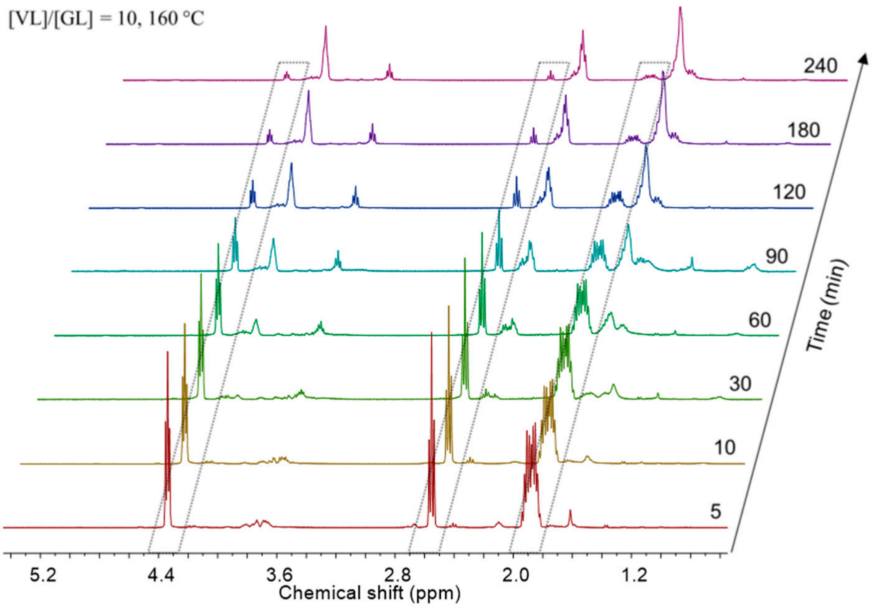

Figure S33 In situ ^{1}H NMR (400 MHz, CD_3OD) spectra of the ROP of VL at 160 °C. Conditions: DMC-NMe = 10 mg, $[VL]_0$ = 11 M, $[VL]_0/[GL]_0$ = 10. Dashed lines denote the monomer signals.

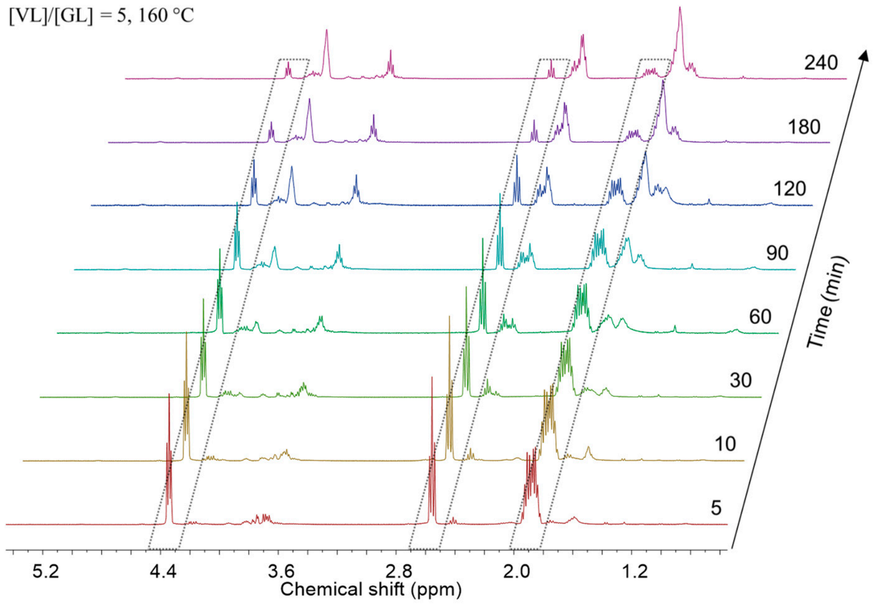

Figure S34 In situ ^{1}H NMR (400 MHz, CD_3OD) spectra of the ROP of VL at 160 °C. Conditions: DMC-NMe = 10 mg, $[VL]_0$ = 11 M, $[VL]_0/[GL]_0$ = 5. Dashed lines denote the monomer signals.

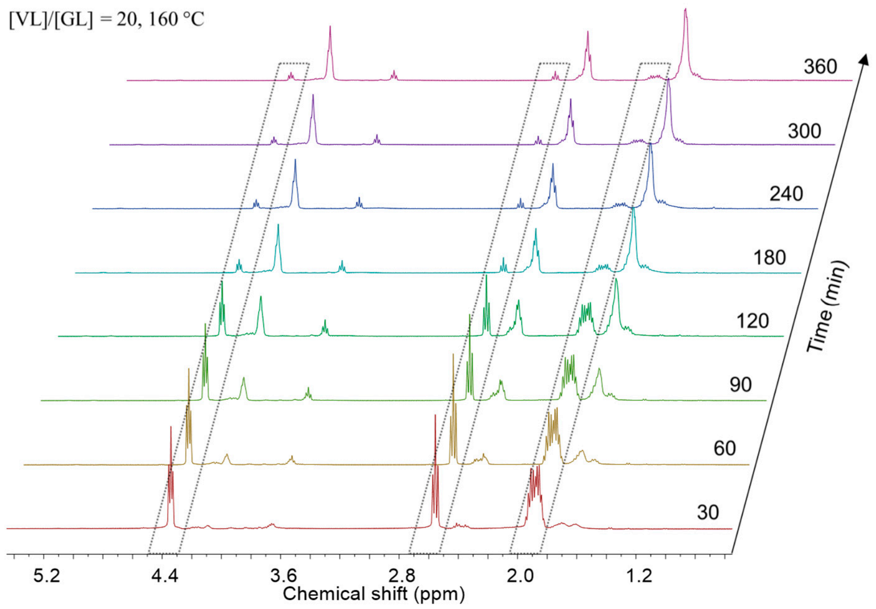

Figure S35 In situ ^{1}H NMR (400 MHz, CD_3OD) spectra of the ROP of VL at 160 °C. Conditions: DMC-NMe = 10 mg, $[VL]_0$ = 11 M, $[VL]_0/[GL]_0$ = 20. Dashed lines denote the monomer signals.

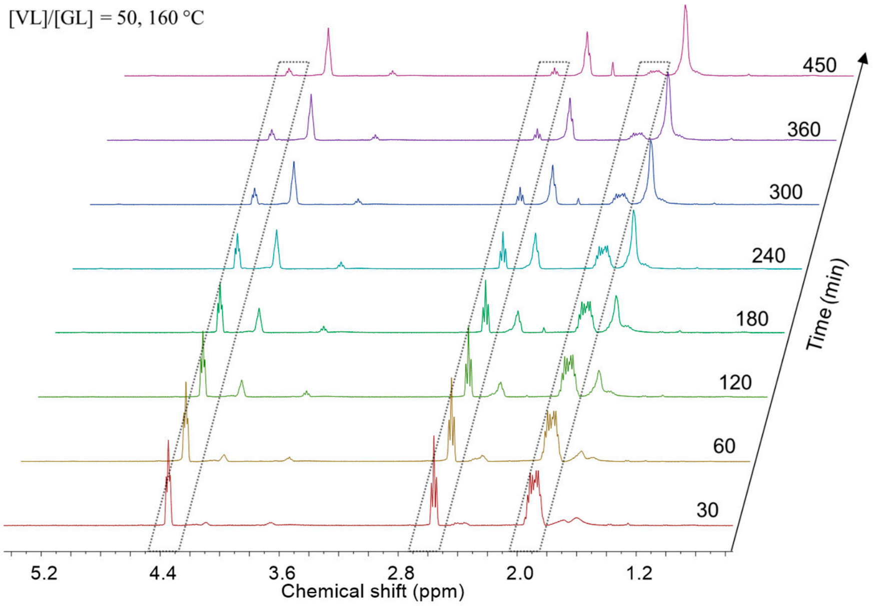

Figure S36 In situ ^{1}H NMR (400 MHz, CD_3OD) spectra of the ROP of VL at 160 °C. Conditions: DMC-NMe = 10 mg, $[VL]_0$ = 11 M, $[VL]_0/[GL]_0$ = 50. Dashed lines denote the monomer signals.

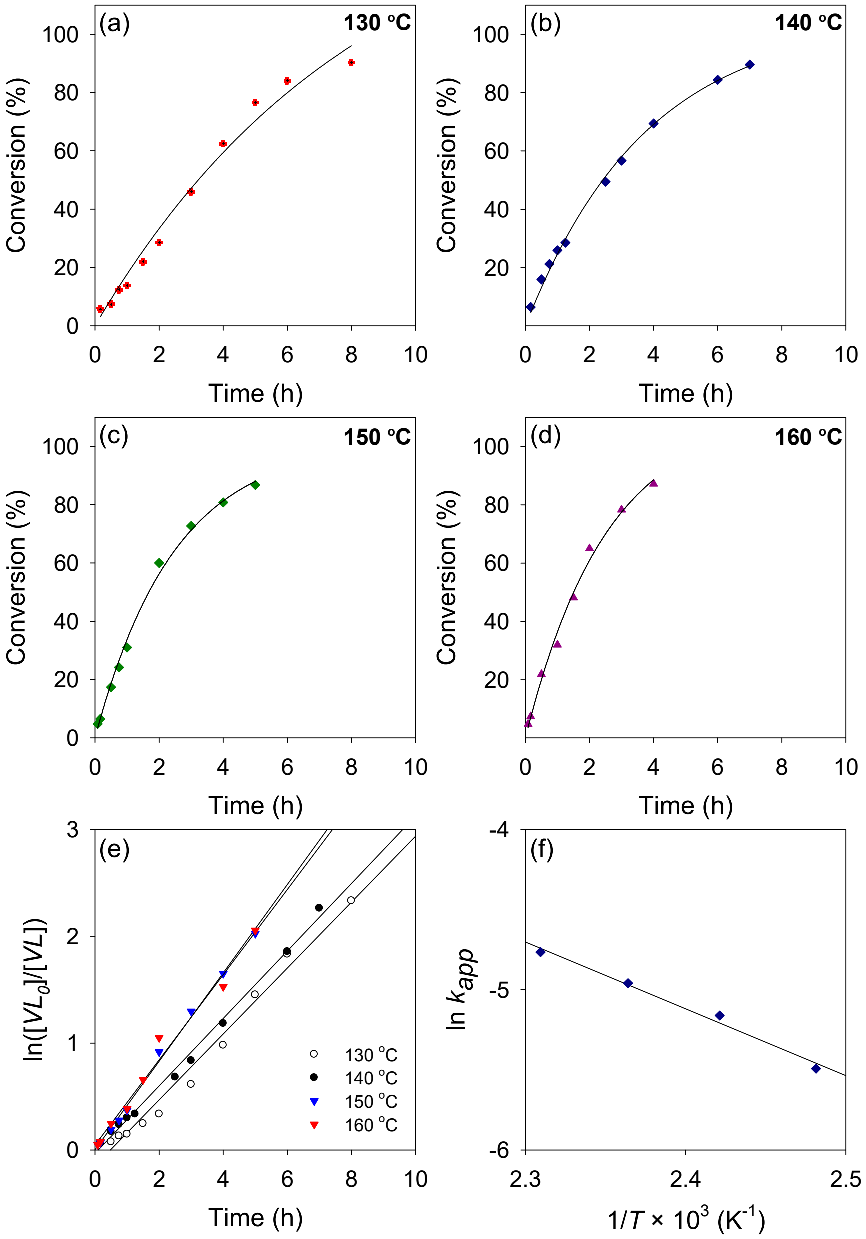

Figure S37 (a)−(d) Plots of monomer conversion versus time obtained at different temperatures, (e) First-order time–conversion plots, and (f) Arrhenius plot of the ROP of VL. Conditions: DMC-NMe = 10 mg, $[VL]_0$ = 11 M, $[VL]_0/[GL]_0$ = 10.

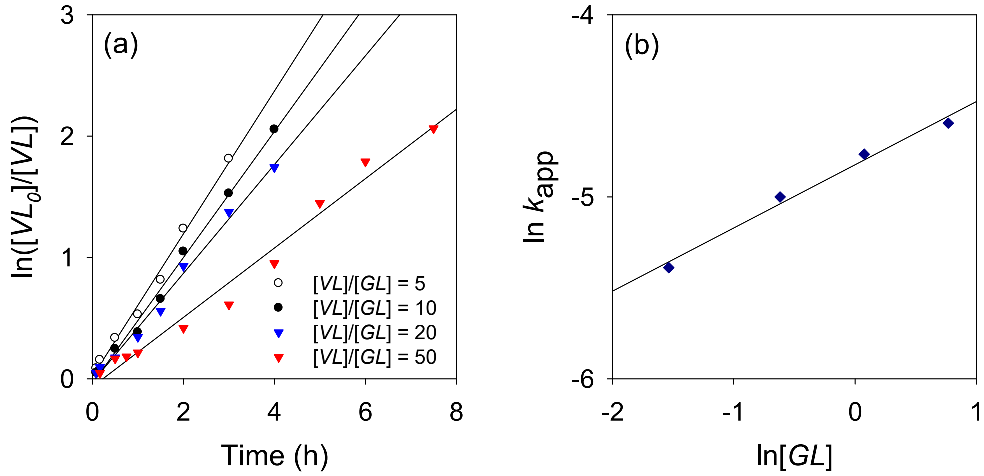

Figure S38 (a) First-order time–conversion plots of the ROP of VL at 160 °C using different amounts of initiator and (b) dependence of the k_{app} on the concentration of initiator [*GL*]. Conditions: DMC-NMe = 10 mg, $[VL]_0$ = 11 M, T_p = 160 °C.

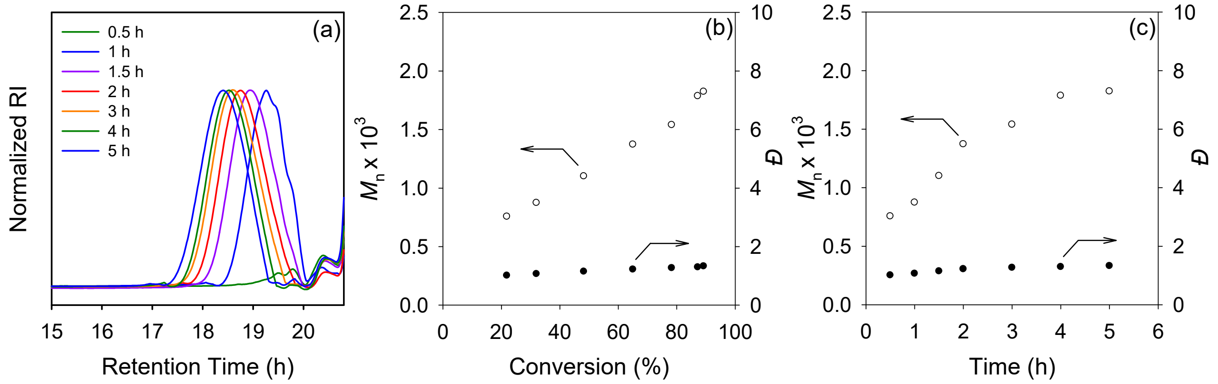

Figure S39 (a) GPC curves of PVL collected at different intervals, plots of M_n and $Đ$ versus (b) monomer conversion and (c) polymerization time. Conditions: DMC-NMe = 10 mg, $[VL]_0$ = 11 M, $[VL]_0/[GL]_0$ = 20, T_p = 160 °C.

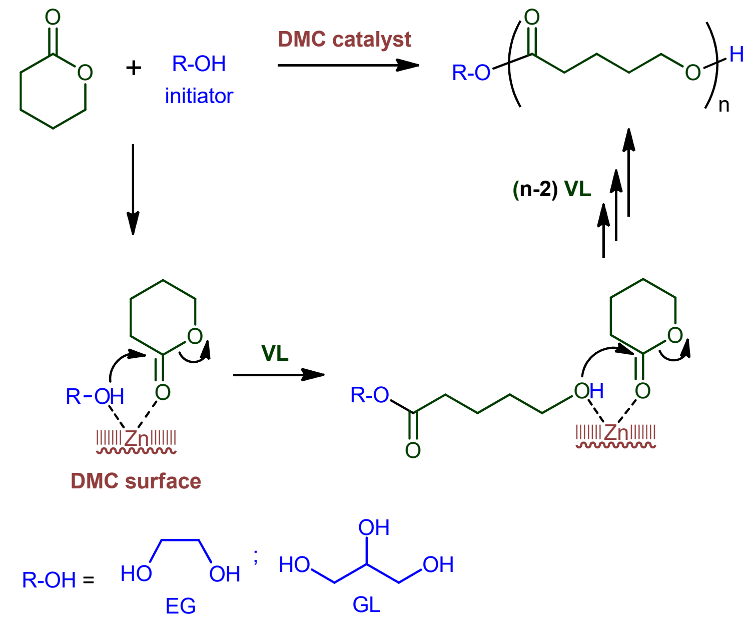

Scheme S1. Plausible mechanism for the DMC-catalyzed ROP of VL via a coordination-insertion pathway.

7. Supplementary tables

Table S1 Summary of the XPS results of Zn 2p spin-orbital components

Catalyst	E_b[b] (eV)	
	Zn $2p_{1/2}$	Zn $2p_{3/2}$
$ZnCl_2$	1046.6	1023.7
DMC-TBA	1046.0	1023.0
DMC-DMAc	1047.5	1024.4
DMC-DMF	1048.0	1025.1
DMC-DMSO	1047.9	1024.8
DMC-NMe	1047.9	1024.9
DMC-NMP	1047.8	1024.7

Table S2 Results for the ROP of VL by DMC-NMe using various $[VL]_0/[I]_0$ ratios

$[VL]_0/[I]_0$	I	t (h)	x_p[b] (%)	GPC	
				M_n	$Đ$
10	EG	6	93	1350	1.21
20	EG	8	94	2000	1.28
50	EG	10	91	3300	1.29
5	GL	4	95	650	1.05
10	GL	6	93	1150	1.15
20	GL	8	90	2040	1.26
50	GL	10	89	4600	1.41
100	GL	12	87	6230	1.57

Conditions: catalyst amount = 10 mg ($[Zn]_0$ = 30 mM), $[VL]_0$ = 11 M, T_p = 160 °C.

Table S3 Results for the copolymerization of PO and lactone using DMC-NMe

Lactone	t (h)	x_p[b] (%)		GPC	
		PO	lactone	M_n	$Đ$
CL	8	99	70	11060	1.59
VL	8	99	93	20680	1.27

Conditions: catalyst amount = 2 mg ($[Zn]_0$ = 6 mM), PO = 1.8 mmol, lactone = 18 mmol, T_p = 140 °C.
